# The Genus *Lophiolepis* Is at Least as Well Supported as *Afrocarduus*, *Afrocirsium*, and *Nuriaea*. Comment on Moreyra et al. African Mountain Thistles: Three New Genera in the *Carduus*-*Cirsium* Group. *Plants* 2023, *12*, 3083

**DOI:** 10.3390/plants13233399

**Published:** 2024-12-04

**Authors:** Emanuele Del Guacchio, František Zedek, Paolo Caputo, Duilio Iamonico, Petr Bureš

**Affiliations:** 1 Department of Biology, University of Naples Federico II, Via Vicinale Cupa Cintia, 26, IT-80126 Naples, Italy; pacaputo@unina.it; 2 Botanical Garden, University of Naples Federico II, Via Foria, 223, IT-80139 Naples, Italy; 3 Department of Botany and Zoology, Faculty of Science, Masaryk University, Kotlářská 2, 61137 Brno, Czech Republic; fzedek@gmail.com; 4 Department of Environmental Biology, Sapienza University of Rome, Piazzale Aldo Moro, 5, IT-00185 Rome, Italy; duilio.iamonico@uniroma1.it

**Keywords:** Asteraceae, Cardueae, taxonomy

## Abstract

Moreyra and co-authors, in their recent paper published in *Plants*, presented a desperately needed Hyb-Seq phylogeny of the genus *Cirsium sensu lato*. Through their findings, they provided a critical assessment of an earlier proposal of ours to separate *Cirsium* sect. *Eriolepis* and *Cirsium italicum* into the genera *Lophiolepis* and *Epitrachys*, respectively. While we fully respect their right not to accept our proposal, we cannot remain silent to their arguments that not only misinterpret our findings and statements but are often in direct conflict with their own results that actually support our proposal to segregate *Lophiolepis*. In addition, as Moreyra and co-authors did not include *Epitrachys italica* (formerly *Cirsium italicum*) in their analyses; they have no basis for arguing in favour or against our proposal to separate it as a monotypic genus. Finally, we conclude that the genus *Cirsium*, as circumscribed by the above-mentioned authors (i.e., including *Lophiolepis*), is definitively not monophyletic.

## 1. Introduction

Recent phylogenetic investigations [1,2,3,4] have unveiled the polyphyletic nature of a broadly circumscribed genus *Cirsium* Mill. This is especially evident when other traditionally recognized genera—*Carduus* L., *Galactites* Moench, *Lamyropsis* (Kharadze) Dittrich, *Nothobasis* (Cass.) Cass., *Picnomon* Adans., *Silybum* Adans., and *Tyrimnus* (Cass.) Bosc—are to be preserved. In the light of these findings, we proposed [3] the separation of two genera from the species-rich *Cirsium* genus, namely *Lophiolepis* Cass. (encompassing 104 Eurasian and North African species) and *Epitrachys* (DC. ex Duby) K.Koch, consisting of a single Mediterranean species. Later, Moreyra et al. [1] proposed the separation of two Central African genera, namely *Afrocirsium* Calleja, Garcia-Jacas, Moreyra & Susanna (with three species) and *Nuriaea* Susanna, Calleja & Moreyra (with two).

Problems with a clear morphological distinction between *Lophiolepis* and *Cirsium* are mainly related to the intermediate features displayed by *Cirsium vulgare* (Savi) Ten. and have been addressed by Bureš et al. [5]. These authors identified *C. vulgare* as an established intergeneric allopolyploid species and segregated it into the monotypic *Ascalea* Hill [5,6]. It is important to note that the segregation of *Lophiolepis* is supported by both phylogenetic evidence and morphological data [1,3,4], as well as by genomic features [4].

Surprisingly, Moreyra et al. [1] strongly dissented with this choice and argued, somehow polemically, against the generic distinctness of *Lophiolepis*. As we are convinced that, on some occasions, they object to our proposal by using inconsistent or unsupported arguments, we are forced to rebut their objections.

## 2. Results and Discussion

### 2.1. Scopes and Limits of Del Guacchio et al. [3]

**Figure 1:**
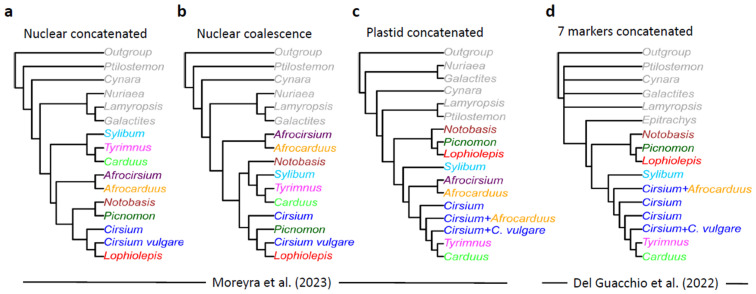
Comparison of phylogenetic trees (simplified and relabeled) from Moreyra et al. (2023) [1] (**a**–**c**) and Del Guacchio et al. (2022) [3] (**d**). In (**d**), each *Cirsium* tip (blue) represents several polytomic branches that we merged into one for clarity’s sake. Only topologies are shown, while branch lengths are arbitrary.

Moreyra and co-authors [1] (p. 13) stated that “[…] the published phylogeny [by [3]] fails to support the classification proposed [by [3]] since *Cirsium s. str.* is not recovered as monophyletic as the entire genus *Carduus* was nested within *Cirsium s. str*. In addition, two species of African *Carduus*, now *Afrocarduus*, are recovered within *Cirsium s. str.* [as intended by [3]], Figure S1, whereas our study reveals that African species of *Carduus*, now *Afrocarduus*, are a group evolutionary [sic!] independent from *Cirsium*”. This is a misleading claim, because we [3] neither proposed a stand-alone *Cirsium* including *Carduus*, nor regarded *Cirsium* (after the exclusion of *Lophiolepis* and *Epitrachys*) as a monophyletic genus; above all, we never proposed any “*Cirsium s. str.*”. In [3], we only proposed a generic recognition of *Lophiolepis* and *Epitrachys* and did not infer any conclusion regarding the monophyly of the group, including the remaining *Cirsium* species. This is obvious throughout our entire contribution, from the title itself [3] (“Towards a monophyletic classification of Cardueae”); from the first sentence of the abstract (“Using molecular data and representative species coverage, we confirmed the monophyly of *Cirsium* sect. *Eriolepis* and, therefore, we propose to treat it as a separate genus (*Lophiolepis*)“); and even from the concluding paragraph of the introduction, which states that “[...] this paper principally aims at the nomenclatural revision of the species belonging to *Cirsium* sect. *Eriolepis* [...]”. Therefore, our work [3] was clearly conceived only as a first step towards a modern classification of *Cirsium s. lat*. We would also like to note that according to our phylogeny [3] (Suppl. fig. S2), the two *Afrocarduus* sequences (retrieved from NCBI) were robustly grouped together but in a poorly supported and resolved clade with a few other *Cirsium* species; this clade, in turn, was nested in a very large and unresolved group including not only *Cirsium* (excluded *Lophiolepis*) but also *Carduus* subg. *Carduus* and *Tyrimnus* (see also Figure 1d). Therefore, on the one hand, our study [3] was doubtlessly not sufficient for any inference about *Carduus* subg. *Afrocarduus* Kazmi and even less for any reassessment of *Cirsium s. lat.* (issues which were never among our aims); on the other hand, the two *Afrocarduus* were confirmed to be not monophyletic with *Carduus* (cf. also in [2]).

In conclusion, we do not understand how our study [3] would support or propose a monophyletic *Cirsium s. str.*, even excluding *Epitrachys* and *Lophiolepis*, or how it would contradict an independent evolution of *Afrocarduus*.

### 2.2. The Separation of Lophiolepis Is Not Based on “Weakly Supported Molecular Results”

Moreyra et al. [1] (p. 2) wrote that “The recent taxonomic proposal for the *Carduus*-*Cirsium* group [by [3]] split *Cirsium* into four genera, accepting Cassini’s subgenus *Lophiolepis* at the generic level, reinstating the genus *Epitrachys* (DC. Ex [!] Duby) K.Koch and describing the hybrid genus x*Lophiocirsium* Del Guacchio, Bureš, Iamonico & P. Caputo, mainly based on the weakly supported molecular results from Ackerfield et al. (2020)” and again [1] (p. 13) “Recently, the authors of [[3]] split *Cirsium* into four genera (*Cirsium s. str.*, *Lophiolepis*, *Epitrachys* and *Lophiocirsium*). This work encompasses a remarkable number of species (*n* = 225), yet it is based only on two nuclear and five plastid markers that have proven to be insufficient for phylogenetic resolution in this genus [[2]].” 

First of all, we take issue with the claim that we split *Cirsium* “into four genera”. In fact, x*Lophiocirsium* is a nothogenus and cannot be included in this count; in addition, we simply proposed [3] the separation of *Cirsium* sect. *Eriolepis* and *Cirsium italicum* DC. at a generic rank from a polyphyletic *Cirsium s. lat*. The proposal was not even new, because *Lophiolepis* (and in a certain sense, *Epitrachys*) had already been recognized at a generic level in the past, e.g., [7,8,9].

Second, five authors [2], who are also co-authors of [1], used only six markers (two nuclear and four plastid); therefore, [2] simply could not prove the insufficiency of the seven markers we used in [3,4], despite what stated by Moreyra et al. [1].

Third, one of the key results of [2] (p. 722, Figures 2 and 3) was a clear phylogenetic delimitation of the so-called “clade one”, comprising a monophyletic “*Eriolepis”* (= *Lophiolepis*) and the two monotypic genera *Nothobasis* and *Picnomon*. The unexpected placement of *Cirsium vulgare* and *C. cephalotes* in “clade two” in [2] (i.e., *Carduus* + the *Cirsium* species not included in *Lophiolepis*) and not in “clade one” [2] actually does not challenge the monophyly of *Lophiolepis* in any way. In fact, *C. vulgare* has been suggested [1,4] and later proven [5] to be of hybridogenous origin, deriving from a cross between *Lophiolepis* and *Cirsium*, so its incongruent position in the phylogenetic trees is fully justified. Regarding the “*C. cephalotes*” specimen employed by [2], we suggest that it was likely misidentified, because in subsequent studies, the newly sequenced individuals of *C. cephalotes* were nested within *Lophiolepis* [3,4], which also happened in the Hyb-Seq phylogeny by Moreyra et al. [1] themselves.

Fourth, our phylogeny [3] was based on 905 sequences, of which 95 (10.50%) were generated by [2], 107 (11.82%) by the group of the same leading authors as [1,2], and 289 (31.93%) from other sources (all were downloaded from NCBI), while 414 (45.75%) were newly generated by our research groups. The tree in [3] includes 255 tips covering 236 taxa and 225 species, while that in [2] includes 173 tips covering 164 taxa and 154 species. Taken together, the claim of Moreyra et al. [1] that our results [3] are “mainly based on the weakly supported molecular results from Ackerfield et al. (2020)” is therefore inaccurate and misleading.

Fifth, Moreyra et al. [1] (pp. 13) further argue that “[...] according to the Supplementary Material [by us [3]], Table S2, the matrix lacks almost 50% of sequences (880 out of 1785)”. This observation, correct per se, may seem to captiously insinuate that our phylogeny has been heavily affected by missing sequences. Actually, using datasets with even far more than 50% of missing data is a common practice [10]. More importantly, the IQ-tree version v2.1.3, which was used in [3] for tree construction, systematically accounts for missing data by implementing terrace-aware computational approaches, and if a problem is found, users are advised to gather more data or filter out gappy taxa/loci [11].

Moreover, despite the statements by [1], our topology [3] largely concurs with their plastid phylogeny (Figure 1c,d), which would be obvious if the *Lophiolepis* clade is labelled as such in the trees, something that Moreyra et al. [1] decided not to carry out in their figures. This similarity seems final to us in order to reject any alleged inadequateness of our results to reach the proposed aim of our previous study, i.e., to characterize the *Lophiolepis* clade.

In conclusion, the separation of *Lophiolepis* from *Cirsium* has been clearly supported in an independent fashion by [2,3], as well as by Moreyra et al. [1] themselves (Figure 1).

### 2.3. There Is No Evidence of an Extremely Frequent Hybridization Between *Cirsium* and *Lophiolepis*

Moreyra et al. [1] (p. 13) stated that according to [3,4] “hybrids between the two largest new genera [i.e., *Lophiolepis* and *Cirsium*] proposed in [[3]] are extremely frequent” and employed this argument against the separation of *Lophiolepis* from *Cirsium*.

In [3], we made the following comment on intergeneric hybridization: “Numerous intergeneric hybrids have been described between *Lophiolepis* and *Cirsium* (especially *C. vulgare*)”. Although we admit that the word “numerous” is ambiguous, we intended to refer to the effectively frequent records of presumed intergeneric hybrids (partly listed in [3]). In fact, we did recombine only one name, because most of the remaining ones were misapplied to taxa which are not of hybrid origin or which require further study. Indeed, later [5], we recognized only four intergeneric hybrids. In [4], hybridization is only mentioned as a process detectable by flow cytometry. It is to be noted, however, that most of the presumed or ascertained hybrids actually involve *Cirsium vulgare* as a parent, which is now recognized as an established hybrid between *Lophiolepis* and *Cirsium* lineages (see discussion in [5]). Furthermore, we have demonstrated [5] that *Cirsium vulgare*, i.e., *Ascalea lanceolata* (L.) Hill, hybridizes with species of the parental lineages (i.e., *Lophiolepis* and *Cirsium*) more frequently than these genera do with each other, as is expected as a consequence of its intergeneric hybridogenous nature. In addition, the propensity to form intergeneric hybrids between *Lophiolepis* taxa and the remaining *Cirsium* species is significantly lower than the propensity to form interspecific hybrids within these genera ([5], Table 1). Although the results of [5] were not available to Moreyra et al. [1], the claim of [1] that “hybrids between the two largest new genera [*Lophiolepis* and *Cirsium*] [...] are extremely frequent” while citing [3,4] is an overinterpretation of what those studies actually say. Moreover, Moreyra et al. [1] do not take into account that these intergeneric hybrids are overall rare and ephemeral. Moreover, although intergeneric hybrids are also reported, for example, between *Cirsium* and *Carduus* (see [12] (p. 206), [13,14,15,16]), this was never used as an argument against the taxonomic distinctness of these genera.

### 2.4. The Carduineae Treatment by Moreyra et al. [1] Is Inconsistent and Contradictory

A relevant inconsistency of the treatment by Moreyra et al. [1] is their choice of keeping together *Lophiolepis* and *Cirsium* as subgenera but segregating the small genera *Afrocarduus* and *Afrocirsium* at the same time. Incidentally, this latter choice is acceptable in our opinion but falls in contradiction with the former. In fact, *Afrocarduus* and *Afrocirsium* (a) are sister groups in each phylogenetic reconstruction published by [1], and (b) can be morphologically separated chiefly (or exclusively!) on account of their phyllary appendages (absent vs. fimbriate) and, less clearly, by characters of the pappus bristles (scabrid-short barbellate vs. plumose) and stems (unwinged or interruptedly winged vs. winged). On the contrary, Moreyra et al. [1] reject *Lophiolepis* as a distinct genus, but the latter (a) is not the sister group of *Cirsium* in two out of the three topologies of theirs (one nuclear, the other plastidial!), and (b) has been proven to differ morphologically from *Cirsium* by more numerous and relevant characters [3,4,5]. The presence/absence of setae on leaves, for example, is universally recognized as diagnostic between *Cirsium* and *Lophiolepis* (e.g., [17,18,19,20,21,22,23]), and several other morphological, anatomical, phenological, karyological, and genomic differences are known (see [5] for details), even if we agree that a wider sampling is necessary to verify character distribution within the genera.

We also add that according to Moreyra et al. [1], the three species segregated by them into *Afrocirsium* “show distinctive characters such as phyllaries with well-developed pectinate appendages, which are absent in *Cirsium* and in all other genera in the *Carduus*-*Cirsium* group […]. However, we consider the presence of a unique diagnostic morphological character combined with strong evidence of being an independent evolutionary lineage to be sufficient to propose a new genus, *Afrocirsium*”. Actually, such “unique” autapomorphy, i.e., pectinate appendages, occurs, for example, in *Cirsium echinus* (M.Bieb.) Hand.-Mazz., *C. sieversii* (Fisch. & C.A.Mey.) Petr., *C. griffithii* Boiss., *C. swaticum* Petr., and *C. wallichii* DC. (the first two species were also included in the dataset of [1]).

Another inconsistency in Moreyra et al. [1] concerns the treatment of intergeneric hybridogenous species. As already discussed, these authors incorrectly reject *Lophiolepis* also based on the presumed “extremely frequent hybrids” and the occurrence of the established hybrid *C. vulgare* itself [1]. However, their own analyses strongly suggest that hybridization also occurred between *Cirsium* and *Afrocirsium*, namely in the origin of *Afrocirsium straminispinum* (C.Jeffrey) Calleja, Garcia-Jacas, Moreyra & Susanna, i.e., one of the only three species of *Afrocirsium*. This presumed hybrid origin was admitted by them, who, however, did not show any doubt in segregating *Afrocirsium* despite this!

As a further inconsistency, it is to be noted that the phylogenetic reconstructions by Moreyra et al. [1] do not include *Cirsium italicum* (i.e., the only representative of the crucial genus *Epitrachys* in our treatment [3]), which has been proven to clearly differ from the taxa involved in this discussion [3,4].

On the contrary, *Lophiolepis* is a rather homogeneous, even speciose group, which likely originated in mountain environments of western Asia–eastern Mediterranean, where it is most diversified [3] (Supplementary Figure S7) and adapted to seasonal drought and grazing, also under human influence. Its reduction to a subgenus of *Cirsium* does not do justice to its natural history and would not help in understanding the reticulate evolution of Carduineae.

### 2.5. The Genus *Cirsium*, as Circumscribed by Moreyra et al. [1], Is Definitively Polyphyletic as a Consequence of Their Own Results

As said, Moreyra et al. [1] fiercely refuse the recognition of *Lophiolepis* at a generic rank; in doing so, however, they completely disregard their own results, especially those obtained by the plastid dataset, where *Lophiolepis* clearly shows a different origin as compared to other *Cirsium* (even after excluding *Nuriaea*, *Afrocirsium*, and *Afrocarduus*) (Figure 1c). Curiously, this is not apparent from the main body of the text, where Moreyra et al. [1] (Figure 3) showed a concatenated chloroplast tree exhibiting a polyphyletic *Cirsium s. lat*. However, if we examine their Supplementary Figure S2, we find that the *Cirsium* clade most distant from the others in their Figure 3 includes all *Lophiolepis* taxa and only them (!). In that reconstruction, *Lophiolepis* resulted as sister to *Picnomon*, and, in turn, both to *Notobasis.* All the remaining *Cirsium* taxa (including *C. vulgare*), represented in their figure by three groups, are paraphyletic, as one of the *Cirsium* clades is a sister to *Carduus* and *Tyrimnus* (Figure 1c).

From what is said above, we may suppose that the maternal lineage of *Lophiolepis* has a last common ancestor with *Picnomon* and *Notobasis*, which is not shared by the paternal line. The latter is largely shared with *Cirsium s. str.*, but evidently diverged over time, as the two clades (i.e., *Cirsium s.str.* and *Lophiolepis*) in nuclear reconstructions are never admixed. This implies that the evolution of *Lophiolepis* has been different and largely independent from that of *Cirsium s. str.*

Therefore, based on a comparison of the incongruent concatenated phylogenies (nuclear and plastidial) in [1], we are forced to conclude that a genus *Cirsium* as conceived by them (i.e., including *Lophiolepis*) cannot be accepted simply because it would not be monophyletic.

### 2.6. Phyletic Classification and Name Inflation

Finally, quoting Moreyra et al. [1]: “...our conservative classification maintaining *Cirsium* as a single genus is also the most robust and operational one, because it avoids the inflation of hundreds of new nomenclatural combinations that would increase the already voluminous synonymy of *Cirsium*”. Nevertheless, this does not prevent these authors from providing several new combinations as well [1]. The dreaded “hundreds of new nomenclatural combinations” (actually 130) had already been provided [3] and are now easily available [24].

In this regard, it might be useful to note that according to the previous contribution of the same group [2], “An alternative solution [i.e., alternative to combine all genera in a single genus] is to recognize each major clade of the phylogeny as a genus. This would result in recognition of seven genera: *Carduus*, *Cirsium*, *Eriolepis* [= *Lophiolepis* in our treatment [3]!], *Notobasis*, *Picnomon*, *Silybum*, and *Tyrimnus*”. The same authors also provided a table with additional morphological differences between *Eriolepis* (= *Lophiolepis*) and *Cirsium* and foresaw a future (at the time) segregation of African mountain thistles. However, from a mere nomenclatural point of view, it is a fortunate case that they refrained from segregating *Cirsium* sect. *Eriolepis* on that occasion, considering that otherwise, they might have provided numerous erroneous combinations under *Eriolepis* [2], entirely neglecting the priority of *Lophiolepis* [25].

## 3. Material and Methods

This response is based on recent achievements in Carduineae taxonomy and evolution [2,3,4,5] and a thorough examination of [1], including their Supplementary Materials. For the sake of clarity, we refer in the text to *Cirsium sensu lato*, the genus traditionally conceived [17,18,19,20,21,22], i.e., including *Lophiolepis*, *Epitrachys*, *Nuriaea*, and *Afrocirsium*; and to *Cirsium sensu stricto*, intending to use the same genus not including those taxa (this, however, would not necessarily imply any assessment about its monophyly). In order to facilitate discussion, in Figure 1, we compared the phylogenetic trees proposed by [1,3], merging monophyletic groups under single labels.

## 4. Conclusions

The *Carduus*-*Cirsium* group presents a substantial phylogenetic challenge. The path to a taxonomic solution to this vast problem necessarily requires successive steps, some of which have already been taken. A full taxonomic resolution of *Cirsium s. lat.* may only arise from greater coverage and the application of phylogenomic techniques, as proposed by Moreyra et al. [1]. We believe this team of colleagues, whose experience and great results we sincerely appreciate, will succeed.

Their concept of keeping a wide concept genus *Cirsium* [1] is altogether respectable. Still, the only reliable alternative to the recognition of the genus *Lophiolepis* is merging it into *Picnomon*, a possibility already discussed but discouraged by us [3]. This option, if adopted, would inevitably lead, for coherence, to merging several small but well-recognized genera, including *Afrocirsium* and *Afrocarduus*.

In addition, to justify a widely circumscribed *Cirsium*, we would be forced to adopt several infrageneric taxa, presumably at different ranks, the meaning of which would be difficult to explain in a modern phylogenetic taxonomy. Moreover, any rejection of taxonomic novelties in favour of nomenclatural stability cannot disregard the idea that genera must be monophyletic. Indeed, this casts doubt on whether, for stability and simplicity purposes, the best choice would be merging again all the above-cited genera in the Linnaean *Carduus* (cf. “solution one” in [2]).
